# Relationship between sperm protamine deficiency and apoptosis in couples with unexplained repeated spontaneous abortions

**Published:** 2016-03

**Authors:** Ali Reza Talebi, Farzaneh Fesahat, Esmat Mangoli, Jalal Ghasemzadeh, Maryam Nayeri, Fatemeh Sadeghian-Nodoshan

**Affiliations:** 1 *Infertility Research Center, Shahid Sadoughi University of Medical Sciences, Yazd, Iran.*; 2 *Faculty of Pharmacy, Shahid Sadoughi University of Medical Sciences, Yazd, Iran.*; 3 *Department of Biology, Islamic Azad University of Ashkezar, Ashkezar, Iran.*; 4 * Stem Cell Biology Research Center, Shahid Sadoughi University of Medical Sciences, Yazd, Iran.*

**Keywords:** *Sperm*, *Apoptosis*, *Protamine*, *Recurrent spontaneous*

## Abstract

**Background:**

Etiology of more than half of Recurrent Spontaneous Abortion. The etiology of more than 50 percent of Recurrent Spontaneous Abortions (RSA) cases has been remained unexplained. It is supposed that RSA may have "paternal effect" due to supply 50% of embryonic genomic content by male gamete.

**Objective:**

The aim of present study was to evaluate the role of sperm apoptosis and protamine deficiency at same time in RSA cases.

**Materials and Methods:**

Forty fertile (control) and 40 unfertile men with RSA (case) were enrolled in this case-control study. Semen analysis was performed in accordance with WHO criteria and sperm apoptosis and protamine deficiency were evaluated by cell apoptosis detection kit and chromomycin A3, respectively.

**Results:**

Results showed significant different between normal morphology and total motility in two groups. Case group had higher percentage of spermatozoa with protamine deficiency and apoptosis compared to controls significantly.

**Conclusion:**

Our results showed that in cases of RSA, in addition to abnormal sperm parameters, we have a high percentage of spermatozoa with protamine deficiency and apoptosis and these two anomalies may consider as important causes of idiopathic recurrent abortions. It should be advised that sperm chromatin and DNA examinations are useful tools in the process of RSA treatments.

## Introduction

The importance of sperm DNA integrity in almost all stages of reproduction have been shown in many studies. Sperm DNA damage is associated with diminished semen quality, fertilization rates, cleavage rates, embryo quality and pregnancy loss (1-5). Because of sperm DNA contributes half of the embryo genomic material, any abnormalities of chromatin/DNA can lead to failures in the reproductive process. Three main molecular causes, namely defective sperm chromatin packaging, apoptosis and oxidative stress are considered as mechanisms of sperm DNA damage. Any abnormalities in these steps may cause sperm DNA damage (6, 7). Oxidative stress upon spermatozoa is induced by an increase in amount of Reactive Oxygen Species (ROS) that are present in the fluids filling the male genital tract (8). It affects the integrity of sperm chromatin causing high frequencies of single and double strand breaks, base modifications, production of base free sites, deletions, frameshifts, cross-links and chromosomal rearrangements (9). Recently, the role of apoptosis in male fertility related to ejaculated spermatozoa have been focused by experts (10, 11). Moustafa *et al* stated that there is a significant correlation between the DNA fragmentation index and apoptosis in spermatozoa of infertile men (12). 

The "Recurrent Spontaneous Abortion" (RSA) or “Recurrent Pregnancy Loss” (RPL) is expressed as three or more successive pregnancy failures before 20 weeks of gestation (13). Although, recurrent abortions may be associated with anatomical, genetic, endocrine, psychological, thrombotic, infectious and immunological causes, more than half of the cases remain unexplained, even following extensive evaluations (14). It is supposed that RPL may have a "paternal effect", because of male gamete supplies 50% of genomic content to embryo (15, 16). "paternal effect" is a new term, which indicates cases where normal pre-implantation embryos are formed; but they fail to implant or are lost soon after clinical pregnancy. 

Regarding three main mechanisms of sperm DNA damage, we have shown the importance of first cause, which was the aberrant sperm chromatin remodeling in couples with idiopathic abortions. In other word, our previous study showed that men from RSA couples have poorer sperm DNA integrity and chromatin condensation than fertiles (17). The data on roles of apoptosis as the second cause of sperm DNA damage in the etiology of RSA are limited. So, the aim of present study was to evaluate the sperm apoptosis and protamine deficiency at the same time and the possible relationship between them in spermatozoa of men from couples with unexplained recurrent abortions. 

## Materials and methods

**Sample** **s** **collection**

In this case-control study, semen samples of 40 fertile normal men without any abortion as control (group B) and 40 men with at least three consecutive pregnancy losses as case group (group A) were collected. The mean value of age was 35±6 in both groups. All samples were selected randomly. This study was approved by the Ethics Committee of Yazd Research and Clinical Center for Infertility and informs consent were obtained from all participants.

A complete evaluation for etiology of recurrent abortion, including sonography, physical examination, cytogenetic, immunological and reproductive hormonal assays was done for case group. Men with varicocele and heavy smokers were excluded from the study. Cases with normal range of mentioned tests were considered as idiopathic RSA patients. 

**Sperm analysis**

Semen samples were collected by masturbation after 2-7 days of sexual abstinence from both groups. Each sample was allowed to undergo liquefaction, and then was evaluated for sperm motility, concentration, viability, and morphology according to World Health Organization (WHO) criteria (18). Briefly, progressive motility, including rapid grade a, slow grade b and non-progressive motility, grade c, were assessed manually by counting 200 spermatozoa. Papanicolaou staining was performed for sperm morphology and sperm count was assessed by Mackler chamber (Sefi Medical Co., Haifa, Israel). Duplicate counting was performed for all analyses by an blinded experienced technician to the study.

**Sperm apoptosis**

The sperm apoptosis was determined by cell apoptosis detection kit (Roche Applied Science, Mannheim, Germany) (19). In this method, the smears were fixed with 4% para-formaldehyde in PBS for 1 hr at room temperature. After washing with PBS, the samples were incubated with 0.3% H_2_O_2_ in methanol for 1 hr to quench endogenous peroxidase activity. The cell permeability was performed with 0.1% Triton ×-100 (Sigma Aldrich Company,St. Louis, USA) at 4^o^C for 5 min, and then incubated with the TUNEL reaction mixture (50 µl) in a humidified chamber at 37^o^C for 1 hr. 

After washing in PBS, they stained with 50 µl converter-POD at 37^o^C for 1 hr. Samples were washed in PBS and exposed to the DAB (3, 3-diaminobezidine tetra hydrochloride) (Roche Applied Science, Mannheim, Germany) substrate solution for color development in a dark chamber at room temperature for 10 min. Finally, the samples were dehydrated in ethanol, cleared by xylene, mounted by DPX and then evaluated by light microscope under 100× eyepiece magnification. In each sample, at least 200 sperm nuclei were counted and repeated again. For negative controls, instead of the TUNEL reaction mixture, slides were incubated with 50 µl of label solution (without terminal transferase).

**Sperm protamine deficiency**

Chromomycin A3 is a fluorochrome specific for guanosine cytosine-rich sequences and is used for evaluation of sperm chromatin protamination degree (20). To do this test, the air-dried smears were fixed by Carnoy’solution (methanol/ glacial acetic acid, 3:1) for 10 min at 4^o^C. The slides were stained by CMA3 solution for 10 min at room temperature. After washing, the slides were mounted by DPX, and at least 200 spermatozoa were counted under 100× eyepiece magnification fluorescent microscope with 460-nm filter. The percentage of spermatozoa with bright yellow heads (CMA3+) and without brightness (CMA3-) were determined.

**Statistical analysis**

Results were analyzed using SPSS software 16 for Windows (SPSS Inc., Chicago, IL, USA). After data normalization with Shapiro-Wilks test, Student’s t-test was applied to compare the groups, and term statistically significant was used to denote two-sided p<0.05 for sperm parameters and cytochemical tests. The correlation between apoptosis and protamine deficiency was performed using Pearson correlation coefficient.

## Results

Fertile and infertile men were matched regarding the age of participants. Results of sperm parameters are listed in table I. Siginificant difference has been showed in normal morphology and total motility between two groups. 

**Figure 1:**
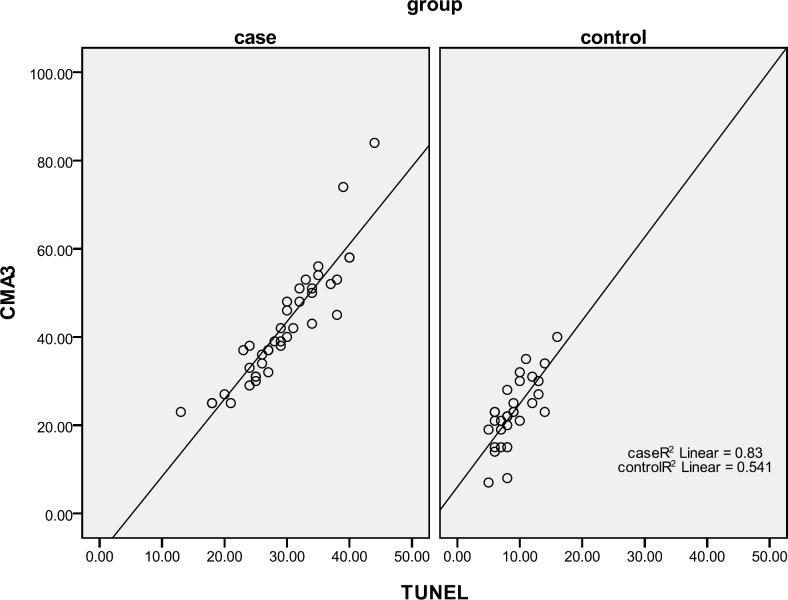
Scatter plot of TUNEL and CMA3 in control and case groups

Table II showed that the percentage of spermatozoa with protamine deficiency and apoptosis is higher in RSA group than control. Significant positive correlation was seen between TUNEL and CMA3 tests in two groups especially in RSA patients (Table III, Figure 1).

**Table I:** Semen analysis in cases (group A) and controls (group B

**Variables**	**Case (group A)**	**Control (group B)**	**p-value**
Count (·10^6^/ml)	87.7 ± 49.7	103.7 ± 50.2	0.21
Rapid motility (%) (Grade a)	17.1 ± 11.5	21.44 ± 10.6	0.26
Slow motility (%) (Grade b)	34.2 ± 8.9	38.41 ± 6.4	0.06
Non progressive motility (%) (Grade c)	14.25 ± 6.15	12.43 ± 5.63	0.59
Immotile sperm (%) (Grade d)	34.45 ± 13.06	27.9 ± 7.38	0.05
Total motility (%) (Grades a, b, c)	65.5 ± 13.06	72.28 ± 7.38	0.0458*
Normal morphology	25.6 ± 11.38	38.6 ± 14.6	0.001

Student’s t-test was applied to compare the case and control groups.

Data are presented as mean±SD.

* Statistically significant (two-tailed), p<0.05

**Table II:** Sperm chromatin/ DNA evaluation in cases (group A) and controls (group B

**Variables**	**Case (group A)**	**Control (group B)**	**p-value**
CMA3	42.35 ± 13.12	23.03 ± 7.5	0.000
TUNEL	30.05 ± 6.3	9.03 ± 2.9	0.000*

Student’s t-test was applied to compare the case and control groups.

Data are presented as mean±SD

* Statistically significant (two-tailed), p<0.05

**Table III:** Comparison of correlation between TUNEL test and CMA3 test

**Variables**	**Case (group A) N=40**	**Control (group B) N=30**
Pearson correlation between CMA3 and TUNEL tests	0.83	
p-value	0.000	0.000

The correlation between apoptosis and protamine deficiency was performed using Spearman's coefficient of correlation

## Discussion

RSA is a complicated problem in field of fertility and there are many studies on its etiology and mechanism with serious controversies. Regard to sperm parameters, just sperm morphology and total motility were indices that showed statistically significant differences between groups. Although, there wasn’t any relationship between sperm morphology and early pregnancy losses in our previous study (20). The RSA patients had abnormal sperm parameters, including motility and morphology (21). Another study suggested an increase in abortion rates in patients with less than 4% of spermatozoa with normal morphology (22).

Present study demonstrated that RSA couples have higher percentage of sperm apoptosis and spermatozoa with protamine deficiency in their samples than controls. Our previous study showed that sperm chromatin condensation and DNA integrity is related to early pregnancy losses and therefore should be assessed in couples with RSA (17). Gupta *et al *also expressed that sperm DNA fragmentation examinations might be appropriate for couples with RPL (23). Our results are in agreement with Bhattacharya, who showed statistically significant difference in rate of sperm DNA damage between men with RSA and men with proven fertility (24).

Recently, Kazerooni and colleagues showed that patients with RPL have more CMA3 and aniline blue positive spermatozoa in comparison with fertile men (21). This study also proposed that poor chromatin quality of sperm maybe considered as a cause of spontaneous recurrent miscarriages. According to our results, protamine deficiency was another characteristic of sperm cells from RSA patients. Since, protamine deficiency and excessive histones are related to each other, and normal histone content of spermatozoa is needed for early embryonic development, we can say that CMA-reacted sperm cells may be considered as one of important factors responsible for RSA. Additionally, in some cases of male factor infertility, there are high percentage of CMA3+ spermatozoa and this test may be used as good predictor of male infertility (25).

In a study conducted by Agarwal and Said in infertile couples; count, motility, and morphology of sperm cells were related to extent of DNA damage (26). They suggested that in many cases, although the DNA damage may not prevent the in vivo fertilization of oocyte, but the resulting zygote fails to get enough growth and may lead to RPL.

The main goal of our study was to investigate the rates of sperm apoptosis in couples with RSA, we showed that these patients have more sperm apoptosis than controls. Although, the apoptosis is considered as the main cause of DNA strand breaks in human spermatozoa, but DNA fragmentation in mature spermatozoa has other origins beside apoptosis. Abnormal chromatin packaging during spermiogenesis and oxidative stress are also considered as the other sources of sperm DNA damage (27). There are several studies indicate the relationship between sperm chromatin anomalies and sperm DNA fragmentation which is critical step in apoptosis (28).

It is also revealed that arrested and fragmented embryos represent the lower implantation rate and high proportion of chromosomal abnormality (2). We showed that both sperm protamine deficiency and apoptosis are seen in semen samples of RSA patients and they are related to each other. In other word, the coefficient of correlation 0.9 between TUNEL and CMA3 tests means that almost all of the protamine deficient spermatozoa are likely to be DNA fragmented. Different methods are used to evaluate sperm DNA damage such as: TUNEL (detecting DNA fragmentation by labeling the terminal end of nucleic acids), Comet (an electrophoresis assay, which evaluates how well the DNA is package within the nucleus), SCSA or sperm chromatin structural assay, which is an assessment of sperm chromatin integrity by measuring the susceptibility of DNA to acid or heat-induced denaturation. 

In the present study, we used TUNEL assay for detection of late stages of sperm apoptosis and CMA3 for detection of sperm protamine deficiency. These techniques are also useful in male fertility assessments and prediction of fertilization, implantation and embryonic development (6, 29). To describe the mechanism of the effects of sperm DNA damage and apoptosis on embryonic development and pregnancy rate, we should know that the paternal genome is only activated 2 days after fertilizationand so, the status of sperm DNA may not dramatically influence the fertilization process (30). 

On the other hand, it has shown that good quality embryos are associated with lower mean percentage of sperm with damaged DNA, presence of higher levels of sperm chromatin damage might impair embryo development (31, 32). Oxidative stress and total antioxidant capacity may be considered as other factors affecting early pregnancy losses (33). Finally, it can be propose that establishment of clinical pregnancy is associated with lower mean percentage of sperm with abnormal chromatin and DNA damage. 

## Conclusion

In conclusion, our results showed that in the cases of RSA, there is high percentage of spermatozoa with protamine deficiency and apoptosis and these two anomalies which are related to each other, may consider as important causes of idiopathic recurrent abortions. It should be noted that sperm chromatin and DNA examinations are useful tools for clinicians in process of infertility in RSA patients treatment.
